# The first imported case of Rift Valley fever in China reveals a genetic reassortment of different viral lineages

**DOI:** 10.1038/emi.2016.136

**Published:** 2017-01-18

**Authors:** Jingyuan Liu, Yulan Sun, Weifeng Shi, Shuguang Tan, Yang Pan, Shujuan Cui, Qingchao Zhang, Xiangfeng Dou, Yanning Lv, Xinyu Li, Xitai Li, Lijuan Chen, Chuansong Quan, Qianli Wang, Yingze Zhao, Qiang lv, Wenhao Hua, Hui Zeng, Zhihai Chen, Haofeng Xiong, Chengyu Jiang, Xinghuo Pang, Fujie Zhang, Mifang Liang, Guizhen Wu, George F Gao, William J Liu, Ang Li, Quanyi Wang

**Affiliations:** 1 Beijing Ditan Hospital, Capital Medical University, Beijing 100015, China; 2 Institute for Infectious Disease and Endemic Disease Control, Beijing Center for Disease Prevention and Control, Beijing 100013, China; 3 Institute of Pathogen Biology, Taishan Medical College, Taian 271000, China; 4 Key Laboratory of Pathogenic Microbiology and Immunology, Institute of Microbiology, Chinese Academy of Sciences, Beijing 100101, China; 5 State Key Laboratory of Medical Molecular Biology, Institute of Basic Medical Sciences, Chinese Academy of Medical Sciences, Beijing 100005, China; 6 National Institute for Viral Disease Control and Prevention, Chinese Center for Disease Control and Prevention, Beijing 102206, China

**Keywords:** hypercytokinemia, imported case, reassortment, Rift Valley fever virus

## Abstract

We report the first imported case of Rift Valley fever (RVF) in China. The patient returned from Angola, a non-epidemic country, with an infection of a new reassortant from different lineages of Rift Valley fever viruses (RVFVs). The patient developed multiorgan dysfunction and gradually recovered with continuous renal replacement therapy and a short regimen of methylprednisolone treatment. The disordered cytokines and chemokines in the plasma of the patient revealed hypercytokinemia, but the levels of protective cytokines were low upon admission and fluctuated as the disease improved. Whole-genome sequencing and phylogenetic analysis revealed that the imported strain was a reassortant comprising the L and M genes from lineage E and the S gene from lineage A. This case highlights that RVFV had undergone genetic reassortment, which could potentially alter its biological properties, cause large outbreaks and pose a serious threat to global public health as well as the livestock breeding industry.

## INTRODUCTION

The first clinical report of Rift Valley fever (RVF) in humans was made in an area near Lake Naivasha of the Rift Valley province in Kenya in 1930.^1^ RVF epizootics and epidemics in livestock and humans have periodically occurred and were geographically restricted to sub-Saharan Africa, but since 2000, this disease has spread to the Arabian Peninsula.^2^ Rift Valley fever virus (RVFV) infection is correlated with several risk factors, including contact with sick animals or contaminated products or exposure to virus-carrying mosquitoes.^3^ Sero-epidemiology revealed anti-RVFV IgG antibodies among livestock and human in countries such as Djibouti where RVF outbreaks have never been reported in either humans or animals,^4^ suggesting the presence of subclinical virus circulation in non-epidemic areas.

Although RVFV has been described in an Angolan returning from South Africa,^5^ and circulating RVFV was reported among animals and humans in other Central African countries such as Central African Republic,^6^ no epizootic or epidemic occurrences have been reported in Angola. Herein, we describe the first case of imported RVFV infection from Angola to China. The longitudinal observation of the clinical manifestations and pro-inflammatory immune mediators of this severe case were reported, and our phylogenetic analysis revealed the virus to be a novel reassortant between lineages E and A.

## MATERIALS AND METHODS

### Laboratory diagnosis

On day 7 after initial presentation of symptoms in Angola, the patient returned to Beijing, and total RNA was extracted from 140 μL of blood and saliva samples using the QIAmp Viral RNA Mini kit (Qiagen, Hilden, Germany) according to the manufacturer's instructions. Using a conventional real-time PCR (RT-PCR) by using Agpath-ID One-Step RT-PCR kit (Thermo, Carlsbad, CA, USA) assay, yellow fever, malaria and chikungunya fever were excluded. Dengue fever, haemorrhagic fever with renal syndrome and viral hepatitis A to E were excluded based on the results of enzyme-linked immunosorbent assays (ELISAs, the antigen and antibody detection kits were from Panbio, Windsor, Australia and Wantai, Beijing, China). The serum sample and saliva sample were both positive for RVFV based on the results of the Agpath-ID One-step RT-PCR kit (ABI, San Francisco, CA, USA), which targets the M segment of RVFV (forward primer: 5′-AGG AAC AAT GGA CTC TGG T-3′, reverse primer: 5′-TTC TTA CTA CCA TGT CCT CC-3′, probe: 5′-AGC TTT GAT ATC TCT CAG TGC CCC A-3′).

### Inflammatory mediator tests

We collected plasma samples from the patient daily since hospitalization and measured the levels of different cytokines and chemokines (Supplementary Table S1) using the Bio-Plex Pro Human Cytokine Array 27-Plex Group I and 21-Plex Group II Kits on a Luminex200 Multiplexing Instrument (Merck Millipore, Darmstadt, Germany) following the manufacturers' instructions. The raw data were analysed using xPONENT 3.1 software (Merck Millipore). Plasma from seven healthy individuals were used as controls.

### Virus genome sequencing and analysing

RNA extracted from blood was used for genome sequencing. Whole genome sequencing was performed using an Ion Torrent PGM Platform (Thermo Fisher Scientific, San Francisco, CA, USA). No readouts corresponding to other haemorrhagic fever viruses were found. All of the RVFV nucleotide sequences available from GenBank were downloaded, and only those that were nearly full length (>85%) were used. This left three datasets corresponding to the L (*n*=107), M (*n*=115) and S (*n*=171) gene segments, which were aligned using Muscle^7^ and then manually adjusted. Phylogenetic analysis was performed on the three single gene segments and the four coding gene regions using RAxML.^8, 9^

The NCBI accession numbers for the L, M and S segments of RVFV are KX611605, KX611606 and KX611607, respectively.

## RESULTS

### Patient history

The 45-year-old patient presented a fever (38.3 °C) associated with chills, malaise, headache, myalgia and large-joint arthralgia on day 1 of disease onset (16 July 2016) in Luanda, Angola. The patient was empirically treated for yellow fever with oral acetaminophen, an intravenous infusion of 5% glucose and a sodium chloride injection at a local hospital. Three days later, his temperature returned to normal, but he developed worsening symptoms of fatigue, malaise, nausea, vomiting, anorexia and severe oliguria. On day 5, he presented midepigastric discomfort and jaundice, and the laboratory tests suggested liver and kidney injury. On the morning of day 7, the patient returned to Beijing and was admitted to Beijing Ditan Hospital.

The patient worked as a forklift worker in Luanda, Angola since February 2014, and lived in the rural district of Luanda. The patient was frequently bitten by mosquitoes. There was no history of contact with livestock or humans with fever, and the patient did not travel out of Luanda since February 2014. Based on the epidemiological data, the most likely source of infection was a mosquito bite.

### Findings on admission

Upon admission to our hospital on day 7, the patient's vital signs were normal (temperature, 37 °C; heart rate, 90 beats per min; respiration rate, 20 per min; and blood pressure, 120/70 mm Hg). He was awake, alert and fully oriented. Physical examination revealed scleral icterus, no splenomegaly or hepatomegaly and no joint tenderness or swelling. Rash and haemorrhagic tendency were absent.

The laboratory tests on day 7 showed renal failure and severe liver damage, with creatinine levels of 1005 μmol/L, blood urea nitrogen levels of 35 mmol/L, total bilirubin levels of 83.8 μmol/L, alanine aminotransferase of 5910 IU/L, aspartate amino transferase of 7570 IU/L and prothrombin levels of 74% (Table 1). Additional abnormal laboratory values included lactate dehydrogenase (1880 U/L), creatine kinase (6680 U/L), and myohaemoglobin (1200 ng/mL; Table 1). The patient was transferred to the intensive care unit because of his presentation of multiorgan dysfunction, including acute kidney injury, acute hepatitis, acute myocardial injury, pancreatitis and rhabdomyolysis.

**Figure 1:**
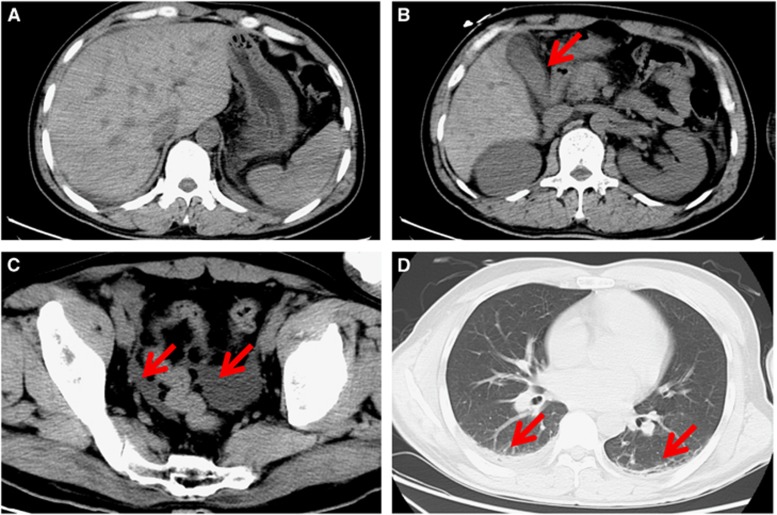
Computed tomography images of the patient on day 7 after clinical presentation of infection. (**A**) Normal morphology and density of the liver, spleen, pancreas and kidney. (**B**) Gallbladder wall thickening (red arrow). (**C**) Fluid in the lower abdominal and pelvic cavities (red arrows). (**D**) A reduced amount of lower pulmonary effusion and pleural effusion (red arrows).

Computed tomography scanning showed pneumonia in the double upper and lower lobes of the lung, pleural effusion, cholecystitis and a small amount of ascites; however, the head CT scan was normal (Figure 1). An ultrasonic cardiogram showed that the left ventricular ejection fraction was 60% and that the heart structure had no obvious abnormalities.

RVFV nucleic acids were detectable in the serum and saliva samples with Ct values of 28.7 and 31.0, respectively, on day 7.

### Clinical course and management

The patient was treated with continuous renal replacement therapy to maintain fluid volume and electrolyte balance. Nausea and pancreatitis limited oral intake, and parenteral nutrition of three litres was given every day with a zero net-volume balance during the first week. For the first three days after admission to the hospital, the patient was also treated with intravenous glycyrrhizinate and reduced glutathione for acute liver injury as well as with methylprednisolone (80 mg/day) to reduce the inflammatory response.

**Figure 2:**
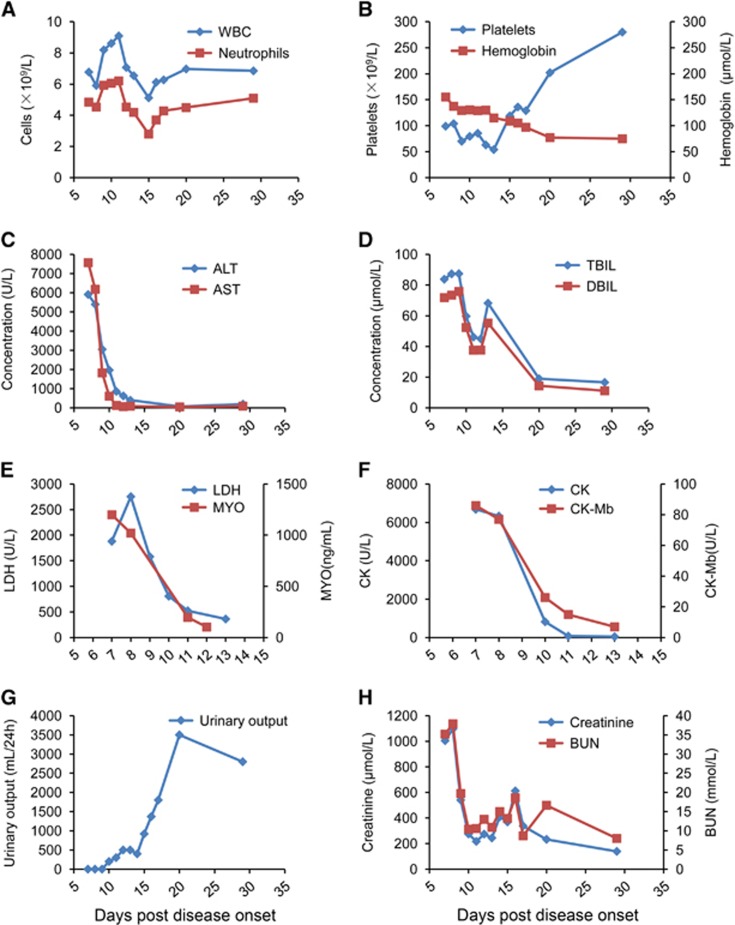
The longitudinal variation of the clinical and laboratory values. The clinical and laboratory variables during the patient's disease progression are shown. (**A**) White blood cells (WBC) and neutrophils. (**B**) Platelets and hemoglobin. (**C**) Alanine aminotransferase (ALT) and aspartate aminotransferase (AST). (**D**) Total bilirubin (TBIL) and direct bilirubin (DBIL). (**E**) Lactate dehydrogenase (LDH) myohaemoglobin (MYO). (**F**) Creatine kinase (CK) and CK-Mb isoenzyme. (**G**) Urinary output per day. (**H**) Creatinine and blood urea nitrogen (BUN).

On day 9, the nausea and vomiting improved, but the anorexia and weakness persisted without further complications. On day 13, his symptoms were ameliorated, and the renal failure and liver injury had improved (Figure 2). He underwent intermittent haemodialysis once every two days starting on day 13. On day 17, the thrombocytopenia improved, and urine output significantly increased (Figure 2). On day 20, the serum creatinine level spontaneously began to decline; thus, the venous catheter was removed. On day 29, his renal function almost recovered, with only the serum aminotransferase levels still slightly elevated (Figure 2). His visual field testing and neurological exam were normal throughout the duration of his hospital stay. Clinical signs and laboratory tests were observed continuously and evaluated daily (Table 1). The patient made a full recovery and was discharged on day 51 (5 September 2016).

**Figure 3:**
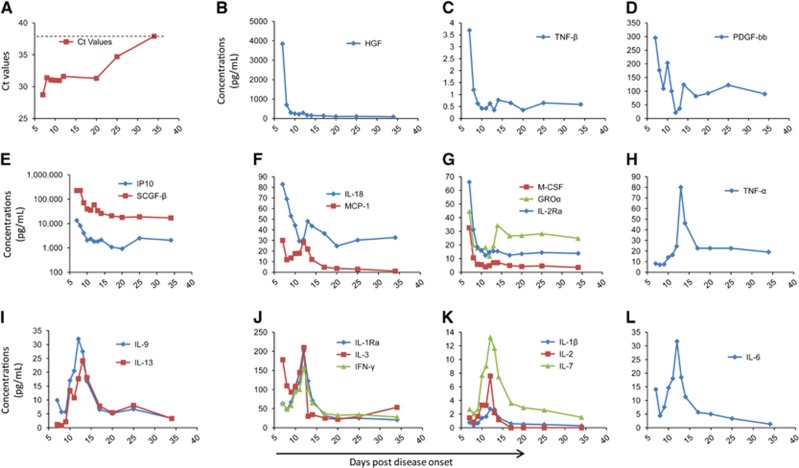
Viremia and hypercytokinemia in the RVF patient. (**A**) The Ct values are based on the RVFV-specific RT-PCR tests by using the blood collected daily from the patient. (**B**–**L**). The longitudinal test of the cytokine and chemokine levels in the plasma from the patient. The daily cytokines and chemokines values are listed in Supplementary Table S1. hepatocyte growth factor, HGF; tumour necrosis factor, TNF; platelet-derived growth factor, PDGF; interferon gamma-induced protein 10, IP10; stem cell growth factor, SCGF-β interleukin, IL; monocyte chemoattractant protein, MCP; macrophage colony-stimulating factor, M-CSF; growth-regulated oncogene, GRO; IL-1 receptor antagonist, IL-1Ra; interferon-gamma, IFN-γ. The concentration of IP10 on day 7 was greater than the highest concentration in the standard curve. Thus, in the 3E, we used the highest point of standard curve (13518.8 pg/mL) as the IP10 concentration on day 7.

To evaluate the viremia of the patient, we used the Ct values from the RT-PCR results of the patient's blood samples; these values are inversely correlated with the viral RNA shedding. The viremia was ameliorated over the first several days with RVFV nucleic acid Ct values of 28.7 on day 7 to 31.4 on day 8. This Ct value was maintained over the following days. From day 20 to day 35, the viremia showed continuous abatement in the patient, and on day 35, the Ct value reached the detection cut-off of the RT-PCR kit for RVFV (38.0, Figure 3A), reflecting the end of the viremia.

### Inflammatory mediators

In general, hypercytokinemia was observed on day 7 after disease onset. Overall, in plasma collected from the patient on day 7, 21 cytokines and chemokines were higher than the upper end of the 95% confidence interval (CI) of the means of the corresponding controls (Supplementary Table S1). Notably, the elevation of the cytokines and chemokines HGF, TNF-β, IP10, SCGF-b, IL-18, MCP-1, M-CSF, GROa and IL-2Ra on day 7 were higher than the upper end of the 95% CI of the healthy controls and showed an obviously rapid decrease after treatment initiation in the following days (Figures 3B, 3C, 3E and 3G). These data confirmed the presence of hypercytokinemia, also known as a ‘cytokine storm', which may have contributed to the immunopathogenesis in the patient. In contrast, the levels of cytokines such as IL-1β, IL-1Ra, IL-2, IL-3, IL-6, IL-7, IL-9, IL-13, IFN-γ and TNF-α, which are correlated with protective immune responses to viruses, were gradually increased in conjunction with the improvement of the symptoms (Figures 3H–3L). The IL-9, IL-13 and TNF-α levels on day 7 were even below the lower end of the 95% CI of healthy controls but increased afterwards (Supplementary Table S1). Starting at day 14, the levels of these cytokines gradually reduced concomitant with the amelioration of viremia in the patient, which may reveal the role of the adaptive immune process. Interestingly, PDGF-bb, which has a significant role in blood vessel formation, showed a generally decreasing trend in the following days after admission, indicating the reduction of the expression of vessel damage factors (Figure 3D).

### Phylogenetic analysis

**Figure 4:**
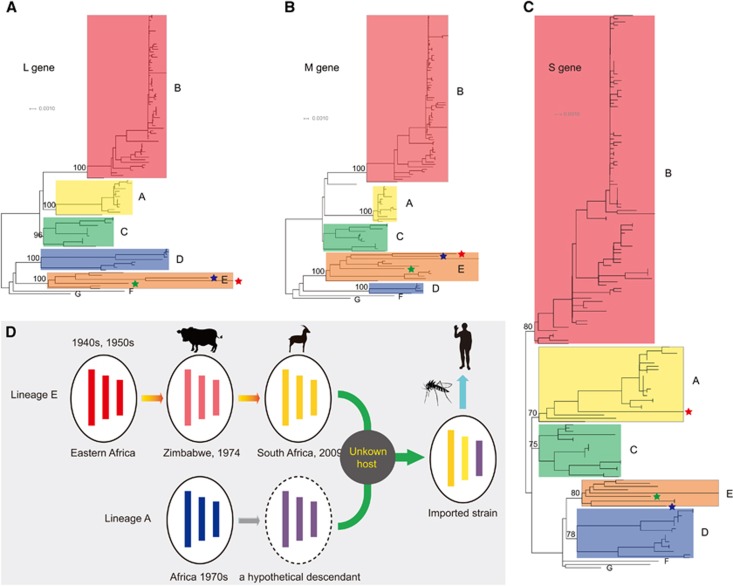
Phylogenetic analysis of the worldwide RVFV gene sequences and the proposed model for the potential source of the first case of imported RVF in China. The phylogenetic trees constructed using the L, M and S gene sequences of the worldwide RVFVs are displayed in **A**–**C**, respectively. In these panels, the red star represents the first imported RVF case in China, the blue star represents the South African strain from sheep from 2009, and the green star represents the bovine strain from Zimbabwe in 1974. (**D**) The proposed model for the potential source of the first imported RVF case in China based on phylogenetic evidence.

Consistent with a previous report,^10^ our phylogenetic trees estimated from the L, M and S gene sequences could also be classified into seven independent lineages (A–G, Figures 4A–4C) despite the inclusion of more newly sequenced strains. In the phylogenetic trees constructed using the L and M gene segments (Figures 4A and 4B, Supplementary Figures S1 and S2), the imported strain fell within lineage E and was closely related to a South African strain (Kakamas) isolated from sheep from 2009. Lineage E also includes several viruses isolated from eastern Africa in the 1940s and 1950s as well as one bovine strain from Zimbabwe in 1974. In the S gene tree, the 2009 South African strain still fell within lineage E close to the bovine strain from Zimbabwe in 1974 (Figure 4C and Supplementary Figure S3). However, the imported strain in China fell within lineage A and was clustered with several strains from Egypt, Madagascar, Zimbabwe and Central Africa from the 1970s, one South African strain from 1981 and one Namibian strain from 2004 (Figure 4C and Supplementary Figure S3).^10^ We then estimated the mean distances of the S gene sequences. In accordance with the phylogenetic analysis, the mean distance between the imported strain and lineage A was 2.5%±0.4%, while that between the imported strain and lineage E was 3.5%±0.4%. We further detected potential recombination events using the complete S gene segment, the non-structural gene region and the nucleocapsid gene region using the Recombination Detection Program (RDP),^11^ respectively, and no positive recombination signals were observed. Therefore, all of the evidence suggested that the imported strain might be a reassortant with the L and M genes from lineage E and the S gene from lineage A.

## DISCUSSION

RVFV can cause symptoms with different severities. Most people with RVF have either no symptoms or a mild illness associated with fever and liver abnormalities. A small percentage of patients develop severe indications including liver failure, renal failure, thrombocytopenia, encephalitis, haemorrhage and miscarriage.^12, 13, 14, 15, 16^ For the first case of imported RVF in China, we used high-volume continuous renal replacement therapy, which treated the renal failure, and methylprednisolone, which reduced the inflammation in the patient.^17^

In previous studies,^18, 19^ the pro-inflammatory mediators IL-1α, IL-1Ra, IL-6, IL-8, IL-10, MIG and IP10 were significantly increased in fatal cases. In our study, 21 cytokines and chemokines in the plasma of the patient were measured upon admission and were higher than the levels in healthy controls, which further demonstrated the contribution of dysregulated inflammatory responses in host individuals to RVFV pathogenesis. The non-structural protein NSs, which is encoded by the S segment of RVFV, has been implicated as the primary virulence factor. NSs can counteract the antiviral effects of the host's type I interferon response.^20, 21^ In this patient on day 7 of disease onset, the levels of cytokines related to the protective immune response (Figures 3H–3L) were equal to or lower than normal levels but gradually increased on day 9 concomitant with the improvement of the disease symptoms. Whether the disordered cytokines and chemokines are associated with the novel genetic constellation of this virus warrants further investigation.

Genetic reassortment of RVFV has been previously reported, and in the present study, we describe a novel RVFV reassortant between lineages E and A. Combined with the phylogenetic evidence and epidemiological data, we propose a model to illustrate the potential source of the imported strain (Figure 4D). RVFV was regarded as originating from eastern Africa and was transmitted to southern Africa in the 1950s.^2^ The descendants of these eastern African strains, such as the Zimbabwe strain (2373/74) and the South African strain (Kakamas), continued to circulate in southern Africa. In the 1970s, another RVFV lineage (lineage A) with a genetic constellation that differed from that of the southern African lineage swept through several African countries and was also transmitted to southern Africa no later than the 1970s. Genetic reassortment might have occurred between descendants of these two lineages in an unknown host somewhere in southern Africa, which would have given rise to the imported strain described here.

It should be noted that the imported strain contains a reassorted S gene segment from lineage A. As mentioned above, the S gene of RVFV is an important virulence factor. Because many strains from lineage A are highly pathogenic and lethal to WF rats^10^ and infected more than 200 000 humans in the 1977–1978 Egyptian outbreak (including 598 fatalities),^2^ we believe that the potential influence of the reassorted S gene on the biological properties of the imported strain requires further study. In addition, whether this reassortment was responsible for the severe clinical outcomes of this patient should be pursued as well.

The patient returned from Angola, a non-epidemic country of RVFV. The subclinical infection results in long-term RVFV circulation in a wide range of hosts throughout Africa, thus increasing the possibility of the accumulation of genetic mutations in the virus via genetic recombination, genetic reassortment and random point mutations. These ‘neglected' viral variants might result in large outbreaks and potentially pose a threat to public health, as we have learned from the 2014–2016 Ebola outbreak in western Africa,^22^ the 2016 Zika virus outbreak in South America and the Caribbean countries^23^ and the 2015 Middle East respiratory syndrome virus outbreak in eastern Asia.^24, 25^ Therefore, vaccines and antiviral agents should be developed in order to control these ‘neglected' tropical diseases.

## Figures and Tables

**Table 1:** Clinical variables and laboratory values during the course of the patient's illness^a^

**Clinical and laboratory findings**	**Day7**^b^	**Day 8**	**Day 9**	**Day 10**	**Day 11**	**Day 12**	**Day 13**	**Day 17**	**Day 20**	**Day 29**	**Normal range**
*Clinical variables*											
Temperature (°C)	37.0	36.8	36.7	37.2	36.9	36.7	37.8	37.7	38.0	37.0	36–37
Mean artery blood pressure (mm Hg)	77	81	89	103	82	102	105	110	97	94	70–105
Urinary output (mL/24 h)	0	0	0	200	300	500	500	1800	3500	2800	1500–2500

*Laboratory values*											
WBC ( × 10^9^/L)	6.77	5.91	8.19	8.61	9.09	7.07	6.54	6.27	6.97	6.85	4.00–10.00
Neutrophils ( × 10^9^/L)	4.84	4.53	5.93	6.06	6.20	4.53	4.19	4.28	4.50	5.10	2.00–8.00
Platelets ( × 10^9^/L)	99	104	70	79.4	85.4	63	54	129	202	280	100–300
Haemoglobin (g/L)	155.4	137.4	129.4	130.4	129.0	130.0	115.0	97.0	77.0	75.0	120.0–160.0
ALT (U/L)	5910	5394	3052	1968	857	622	398	—	67	194	9–50
AST (U/L)	7570	6180	1819	612	125	70	87	—	46	90	15–40
TBIL (μmol/L)	83.8	87.3	87.4	59.7	46.1	45.0	68.2	—	19.0	16.6	0–18.8
DBIL (μmol/L)	71.8	73.4	75.9	52.4	37.6	37.7	55.3	—	14.4	11.1	0–6.8
LDH (U/L)	1880	2754	1580	807	523	—	362	—	—	—	80–285
MYO (ng/mL)	1200	1020	—	—	197	102	—	—	—	—	0–140
CK (U/L)	6680	6332	—	819	74	—	44	—	—	—	38–174
CK-Mb (U/L)	86	77	—	26	15	—	7	—	—	—	<25
Creatinine (μmol/L)	1005	1097	540	275	217	274	244	338	233	139	59–104
BUN (mmol/L)	35.2	37.9	19.7	10.4	10.6	13.0	10.9	8.7	16.6	8.0	1.7–8.3
PT (s)	14.0	14.1	10.3	10.3	10.7	10.8	12.0	11.3	—	10.2	9.4–12.5
AMY (U/L)	132	—	284	275	192	—	—	—	—	—	35–135
LPS (U/L)	58.0	—	468.0	341.0	67.0	—	—	—	—	—	5.6–51.0
CRP (mg/L)	18.5	17.7	—	—	16.1		28.5	—	—	—	0–5.0
PCT (ng/mL)	71.80	—	—	—	—	—	—	6.80	—	—	<0.05

Abbreviations: alanine aminotransferase, ALT; amylase, AMY; aspartate aminotransferase, AST; blood urea nitrogen, BUN; creatine kinase, CK; C-reactive protein, CRP; direct bilirubin, DBIL; lactate dehydrogenase, LDH; lipase, LPS; myohaemoglobin, MYO; procalcitonin, PCT; prothrombin time, PT; total bilirubin, TBIL; white blood cell, WBC.

a Unavailable information for the tests is denoted as '—'.

b Days after disease onset.
