# Supplementary material for: The first imported case of Rift Valley fever in China reveals a genetic reassortment of different viral lineages
Source: Emerg Microbes Infect. 2017 Jan 18;6(1):e4–. doi: 10.1038/emi.2016.136 (PMC5285499; doi:10.1038/emi.2016.136)
Supplement: Supplementary Table 1 [file emi2016136x1.pdf]

Cytokine/chemokine	Patient[b]	Healthy controls, n = 7		Panel in Figure 3
		Mean	95% CI	
CTACK[c]	**378.5**[d]	89.9	51.5-128.2	
Eotaxin	34.1	32.3	20.1-44.5	
FGF basic	14.4	12.0	5.9-18.1	
G-CSF	**26.7**	7.8	5.9-9.7	
GM-CSF	18.6	12.6	4.1-21.1	
GROα	**44.6**	22	8.6-35.5	3G
β-NGF	**3.7**	0.2	0.2-0.2	
HGF	**3844.3**	48.6	27.1-70.2	3B
IFN-γ	**63.8**	51.1	40.5-61.7	3J
IL-10	4.5	3.9	0-7.9	
IL-12p70	1.2	7.4	1.5-13.3	
IL-13	1.2	1.8	1.3-2.2	3I
IL-16	**390.0**	35.4	15.2-55.6	
IL-17	5.2	14.9	6.8-23.0	
IL-18	**82.9**	11.4	6.4-16.4	3F
IL-1β	0.8	0.8	0.5-1.1	3K
IL-1Ra	**63.3**	28.2	17.5-38.9	3J
IL-2	1.5	3.6	1.8-5.4	3K
IL-2Ra	**66.1**	1.2	-0.5-2.9	3G
IL-3	177.8	99.8	39.0-178..3	3J
IL-4	2.2	2.7	2.4-2.9	
IL-5	1.8	2.7	2.1-3.3	
IL-6	**14**	2.0	0.8-3.1	3L
IL-7	**2.7**	2.0	1.3-2.6	3K
IL-8	17.2	25.1	2.7-47.5	
IL-9	9.9	31.7	12.5-51.0	3I
IP10	**OOR >**[e]	206.4	141.1-271.6	3E
MCP-1	**30.0**	7.4	0.9-13.9	3F
M-CSF	32.6	21.7	5.0-38.4	3G
MIF	**190.4**	12.3	8.6-16.1	
MIG	**5041.3**	59.1	37.8-80.4	
MIP-1a	0.6	1.5	0.8-2.1	
MIP-1b	24.7	40.7	29.3-52.1	
PDGF-bb	295.4	502.4	372.3-632.5	3D
SCF	**91.1**	7.4	6.1-8.6	
SCGF-β	**2.26×10^5**	2128.0	1455-2801	3E
SDF-1α	**89.3**	16.1	11.9-20.4	
TNF-α	8.2	10.9	9.0-12.7	3H
TNF-β	**3.7**	0.1	0.1-0.2	3C
TRAIL	**12.5**	7.3	5.4-9.2	
VEGF	6.8	22.9	7.3-38.5	

Supplementary Table S1 The plasma cytokines and chemokines[a] on admission (Day 7 post disease onset).

[a] Levels are given in pg/mL.

[b] The cytokines and chemokines in the plasma collected on the first day of hospitalization (Day 7) were tested.

[c] Abbreviations: CTACK, cutaneous T cell-attracting chemokine; FGF, fibroblast growth factor; G-CSF, granulocyte colony stimulating factor; GM-CSF, granulocyte macrophage colony-stimulating factor; GRO, growth-regulated oncogene; β-NGF, β-nerve growth factor; HGF, hepatocyte growth factor; IFN-γ, interferon-gamma; IL, interleukin; IL-1Ra, IL-1 receptor antagonist; IP10, interferon gamma-induced protein 10; MCP, monocyte chemoattractant protein; M-CSF, macrophage colony-stimulating factor; MIF, macrophage migration inhibitory factor; MIG, macrophage-induced gene; MIP, macrophage inflammatory protein; PDGF, platelet-derived growth factor; SCF, stem cell factor; SCGF-β, stem cell growth factor; SDF, stromal cell-derived factor; TNF, tumor necrosis factor; TRAIL, TNF-related apoptosis-inducing ligand; VEGF, vascular endothelial growth factor.

[d] The cytokines and chemokines that are higher than the upper 95% CI of healthy controls are denoted in bold font.

[e] The "OOR >": Out of range above, which means larger than the highest concentration in the standard curve. The highest concentration of IP10 standard curve is 13518.8 pg/mL.
