# Supplementary figures and images for: The first imported case of Rift Valley fever in China reveals a genetic reassortment of different viral lineages
Source: Emerg Microbes Infect. 2017 Jan 18;6(1):e4–. doi: 10.1038/emi.2016.136 (PMC5285499; doi:10.1038/emi.2016.136)

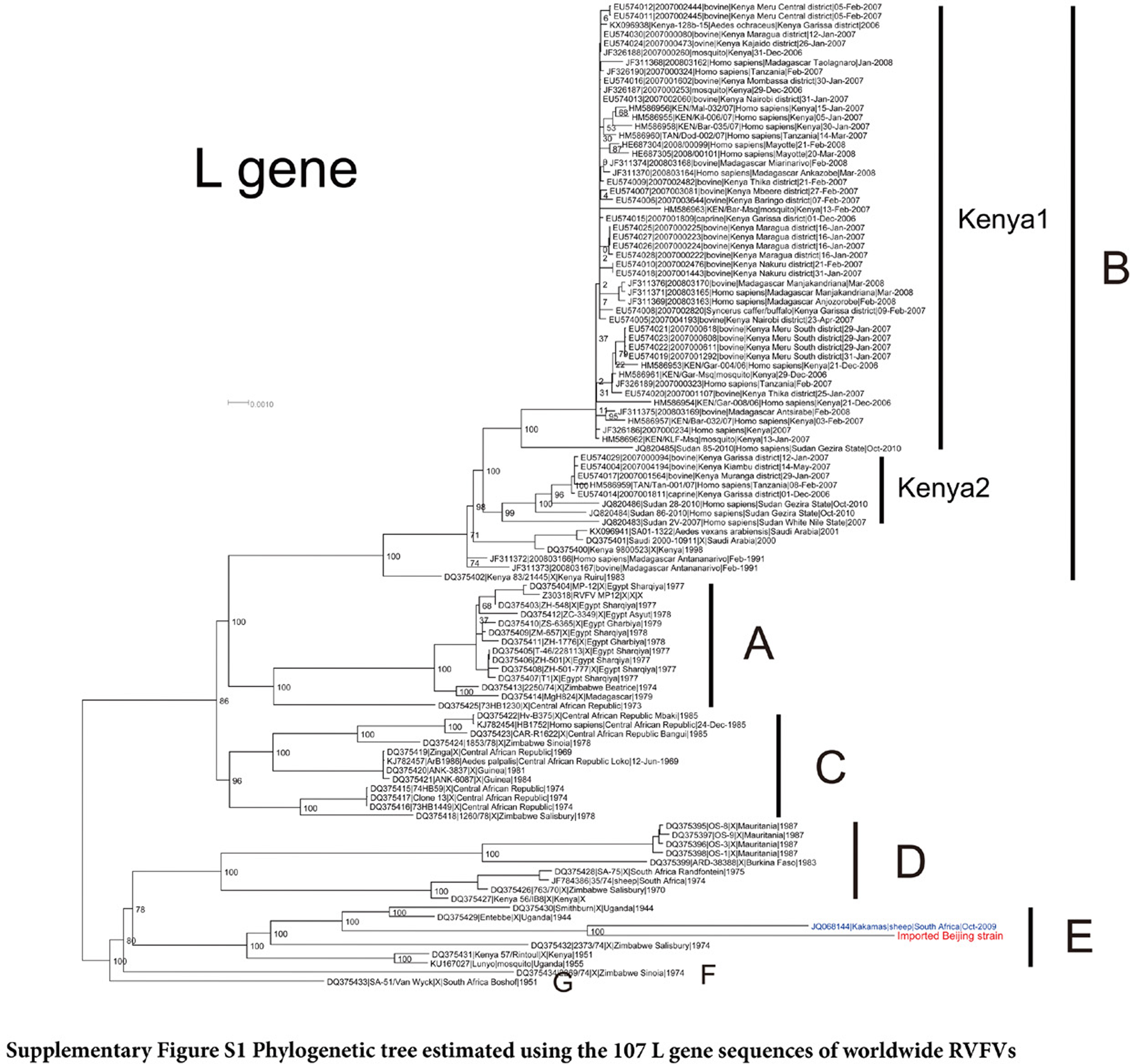

Supplement: Supplementary Figure 1 [file emi2016136x2.tif]
